# Lawless Liberty

**Published:** 1888-12

**Authors:** 


### LAWLESS LIBERTY.

### MORALLY AND PHYSICALLY CONSIDERED.

A fine American writer has said that "the existing evil in our country of youthful rebellion against all wholesome authority, is now universally felt and complained of throughout the land, and that an antagonistic spirit seems to be aroused the moment any restraining influence is excercised." He also remarked that the great want of deference to elders, refusal to heed their words of wise counsel and the absence for veneration of grey hairs, are commented upon by all intelligent foreigners visiting our country—even by the Chinese and Japanese whom we call heathens.

That domestic, social, legal, commercial, and political insubordination to right doing, does exist all over our country to a fearful extent, no intelligent person can deny. It is indeed a great scourge, and is infecting our whole moral economy with a blight so baleful in its influence, it is already deeply felt to the sorrow of our homes, the disgust of our communities, the dishonor of many public organizations, and the grave apprehension of the nation itself. What its effect upon the coming generation will be is fearful, to comtemplate.

This lawless latitude of principle and action, this license to follow one's own devices, and to accomplish one's own unrighteous ends, is called liberty—a shameful perversion and desecration of one of the grandest and most significant words in our language.

A wise and distinguished citizen of our country, whose name I cannot now recall, publicly declared that he would prefer his son should learn self-control and obedience without any other education, than to have all the learning of colleges and universities, with these most important and nobly restraining influences left out of his training and instruction. Obedience to a superior power, superior in its quality as well as its ability, is the first salutary law of the universe; it is the bugle note that calls for order and unity in the ranks of the people, and is the watchword in happy, well regulated and wisely-governed homes, which homes, as has been so often said, are after all the bulwarks of the nation. Prompt and willing obedience to what we might call the law of principle, and the rigid observance of its rules in all places and under all circumstances, is the only way in which to wisely train the youth of a nation and this obedience is just as incumbent upon men in all departments of life as it is upon the boys who come after them.

The results of lawless liberty in thought, word, method or action, when broadly and thoughtfully considered, are absolutely appalling, and they are so pregnant with pernicious and unlimited diffusion, volumes could be written—possibilities for evil, shame, sorrow, dishonor, and utter ruin to body and soul.

The damage to moral character which this disposition to throw off all righteous restraint brings about, is apparent to men of only ordinary intelligence; but even some physicians do not seem to consider the fact that not only morality, but pure and undefiled religion in our daily and hourly life, is absolutely essential to the attainment and preservation of perfect physical and mental health in their most possible vigor, symetry, and beauty, as designed by the Creator of all good. Doctors of medicine should indeed be the oracles in everything that pertains to that grand temple of the mind and soul which we call the body. To truly fill this high office, they know they must study the results of indulgence in every phase of moral and mental emotion, and the condition of home, social and public environments as effecting physical health and long life, as well as the philosophy of meteria medica, etc. The true skilled physician now feels that he is constrained by his very office to lift up his voice so as to be heard all over the land in strong condemnation of all vice, immorality, indulgence in every unrighteous taste and emotion, and license to sin of every kind, because he knows that all these are surely destructive to health and length of life, to the conservation of a pure and wholesome social system, to the happiness and important influence of the homes of the people and to the strength and security of the nation. T. S. P.

---

P. BLAKESTON & SON VISITING LIST 1889. Thirty-eighth year of its publication. Successors to Lindsav & Blakeston, 1012 Walnut St., Philadelphia. Very conveniently arranged for accounts and memoranda. Contains Marshall Hall's method in asphyxia, urinary tests, French and decimal system of weights and measures, dose taflle, new remedies, Posological table, examination of urine, Sylvester's method artificial respiration, etc. Price $1.00 to $3. according to size.

